# Parallelized label-free monitoring of cell adhesion on extracellular matrix proteins measured by single colour reflectometry

**DOI:** 10.1007/s00216-021-03522-1

**Published:** 2021-07-16

**Authors:** Johanna Hutterer, Günther Proll, Peter Fechner, Günter Gauglitz

**Affiliations:** 1 Institute of Physical and Theoretical Chemistry (IPTC), Eberhard Karls University Tuebingen, Auf der Morgenstelle 18, 72076 Tuebingen, Germany; 2 BioCopy GmbH, Elzstrasse 27, 79312 Emmendingen, Germany

**Keywords:** Single colour reflectometry (SCORE), Cell adhesion, Direct optical sensing, Multiplexing, Extracellular matrix proteins, Cell-substrate interaction

## Abstract

The understanding of the initial cell adhesion to biomaterials is crucial for the survival of implants. The manifold possibilities to tailor an implant surface and the diverse requirements for different implant applications necessitate a timesaving and highly parallelized analytical methodology. Due to its intrinsic advantages (label-free, time-resolved, robust against temperature fluctuations, and particularly the multiplexing possibilities), single colour reflectometry (SCORE) is used for the first time to investigate cell adhesion to different extracellular matrix protein–coated surfaces. The excellent correlation between the novel SCORE technology and well-established reference methods proves that the results obtained by using this direct optical method are able to reflect the cell binding processes at the transducer surface. Additionally, the high time resolution of SCORE revealed the differences in the adhesion behaviour of the cells on the different extracellular matrix protein–coated glass slides during the initial adsorption phase and during the spreading of the cells on the surfaces. Therefore, we conclude that SCORE is a perfectly suited methodology for studying the entire cell adsorption process, including morphological changes, and shows great potential for other cell-based sensing applications.

**Graphical abstract:**

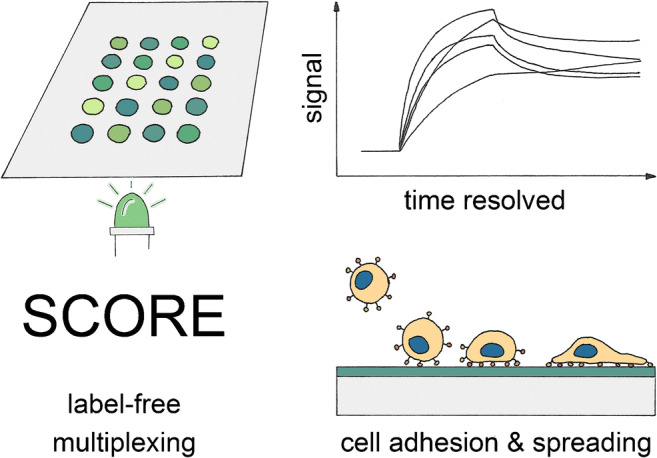

## Introduction

The interactions of human cells with adhesion proteins play a major role in several processes in living organisms including humans. Dysregulation of these processes leads to the development of cancer, fibrosis, cardiomyopathy, arteriosclerosis, and autoimmune and inflammatory diseases [1]. Additionally, the understanding of these processes is crucial for the survival of implants [2]. Huge effort is dedicated to the development of biocompatible and bioactive implant surfaces to tailor a specific cellular response and thereby improve the survivability and performance of the implant. In the early years of this research area, scientists concentrated on finding generic solutions to improve the biocompatibility of implant materials [3]. However, as research progressed, it became clear that each implant requires an individually tailored solution, which necessitated the evaluation of the influence factors on the cellular response for each new application.

Besides the mechanical properties such as material stiffness, surface roughness, and micro- and nano-structuring, a common approach to tailor the surface properties is the adjustment of the hydrophobicity or the chemical composition of the surface [4], the coating with a different material, or the decoration of the surface with bioactive substances [5, 6]. All these methods aim at intervening in the initial contact of the body with the material. The initial adsorption of proteins and other substances in the body fluids can be controlled by adjusting the hydrophobicity of the surface. This change in initial protein adsorption can have huge effects on the cell behaviour [7–9]. Also, coating the implant with other materials often serves to tailor or inhibit the adsorption of unwanted proteins and other substances. To passivate the surface, often hydroxyapatite, metallic oxides, or polyethylene glycol are used [10–12]. However, this process is rather non-selective. A more selective approach is to trigger specific responses of the body by coating the surface with bioactive substances [6, 13, 14].

To assist the adhesion of specific cell types on the implant, often proteins or peptides found in the extracellular matrix (ECM) of the desired cell type are used. While the influence of some ECM proteins—e.g. fibronectin and collagen—on different cell types has been explored extensively, other proteins received less attention in the past [15]. This is probably due to the fact that the investigation and evaluation of these cell adhesion process is a rather complex and time-consuming task.

The analytical methods to study cell adsorption processes on biomaterials are manifold and include, e.g. optical microscopy, direct optical biosensing, impedance spectroscopy, or quartz crystal microbalance. Beside optical microscopy, the direct optical biosensor technologies have proven in the past to deliver valuable analytical insights during cell-based assays [16–20]. Especially photonic crystals [21], imaging surface plasmon resonance [22], and grating couplers [23] even in combination with atomic force microscopy to study single-cell adhesion forces [24] have been used to study cell adsorption or receptor-mediated changes of the cell metabolism caused by the binding of effector molecules. In the latter case, commercial systems based on grating coupler technologies such as EnSpire® Multimode Plate Reader from PerkinElmer or Epic® are used quite frequently. In this specific case, the signal changes are measured as changes of the refractive index within the range of the evanescent field—a process also called as dynamic mass redistribution—caused by receptor activation in Chinese hamster ovary cells [25].

However, for the anticipated assay concept in this study, we had to select a suitable transduction method which allowed a time-resolved investigation of a relatively large surface area (> 1 cm^2^) under flow-through conditions. Furthermore, the method should also be robust against temperature fluctuations. Due to its intrinsic advantages (label-free, robust against temperature fluctuations, and larger penetration depth) compared to other direct optical methods, reflectometric interference spectroscopy (RIfS) [26] and RIfS-based methods proved suitable for measuring cell adsorption [27–31]. Though, it is not easy to achieve a high degree of parallelization of the RIfS technology. However, this would offer the chance for combining multiplexing with time-resolved acquisition of many data points for kinetic evaluation beyond equilibria.

The need for this novel, timesaving, and highly parallelized methodology becomes even more evident when considering the prevalent problem when comparing the results from different cell-protein interaction studies, which is the large number of variations caused by inhomogeneities in the integrin expression in different species, cell donors, cell lines, and culture conditions [15]. Additionally, in cell-based sensing, the use of sophisticated fluidics ensures an equal distribution of the cells across the entire investigated area and allows for a quick and easy exchange of sample fluids.

To tackle these challenges, a new methodology was chosen which adds parallelization to the advantages of RIfS. For the first time, the single colour reflectometry (SCORE) [32, 33] is used to investigate the cell adhesion to differently modified surfaces. Combining the embedded microfluidics, the label-free optical principle, the high time resolution, the robustness against temperature fluctuations, the large penetration depth, and the multiplexing possibility, SCORE is superior to other state-of-the-art methods. We therefore expect SCORE to have the potential to extend the spectrum of technologies available for studying cell adhesion processes. To validate this new approach, we compared the results to the results given by well-established fluorescent microscopy of fixed and stained adherent cells.

## Methods and materials

### Surface preparation

SCORE-compatible glass slides (provided by BioCopy GmbH, Emmendingen, Germany) were cleaned first for 10 min in an ultrasonic bath with washing solution 1 (1 part concentrated ammonia solution, 5 parts 30% hydrogen peroxide, 20 parts milli-Q water, 80 °C), then for 10 min in an ultrasonic bath with washing solution 2 (1 part concentrated hydrochloric acid, 5 parts 30% hydrogen peroxide, 20 parts bidest water, 80 °C), and finally in milli-Q water. Polydimethylsiloxane (PDMS) templates for positioning the respective protein spots were prepared by trimming a 2-mm-thick PDMS sheet (provided by BioCopy GmbH, Emmendingen, Germany) to the dimensions of the observable area in SCORE measurements. At the positions intended for the protein spots, holes were punched into the prepared PDMS sheet using a biopsy tool (diameter of 2 mm). These PDMS templates were cleaned for 5 min in acetone and with 0.5% sodium dodecyl sulfate solution at pH 1.5 in an ultrasonic bath and then thoroughly washed with demineralized water. On each glass transducer, 20 protein spots were placed presenting quadruplets of 5 different ECM proteins. For the spotting of the proteins, fibronectin (F1056, Merck KGaA, Darmstadt, Germany), vitronectin (SRP3186, Merck KGaA, Darmstadt, Germany), collagen IV (C6745, Merck KGaA, Darmstadt, Germany), laminin (L4544, Merck KGaA, Darmstadt, Germany), and albumin (A1653, Merck KGaA, Darmstadt, Germany) were diluted to a final concentration of 6 μg ml^−1^ in phosphate-buffered saline (PBS, 14190094, Thermo Fischer Scientific, Karlsruhe, Germany). After positioning the PDMS template on the glass substrates, 2 μl of the respective protein solutions were applied to the cavities in the PDMS template. The proteins were dried on the glass surface for 25 min at 37 °C.

### Cell culture

Wild-type mouse epithelial fibroblasts (MEF, kindly provided by Tilman E. Schäffer) were cultured in Dulbecco’s Modified Eagle’s Medium (DMEM, D5796, Merck KGaA, Darmstadt, Germany) supplemented with 10% foetal calf serum (A3160801, Thermo Fischer Scientific, Karlsruhe, Germany) and 1% penicillin/streptomycin (15070063, Fisher Scientific GmbH, Schwerte, Germany). At a confluency of 70%, cells were detached by trypsin/EDTA 5% (25300054, Thermo Fischer Scientific, Karlsruhe, Germany), centrifuged at 1000 rpm for 10 min, and resuspended in Leibovitz’s L-15 Medium (21083027, Thermo Fischer Scientific, Karlsruhe, Germany) to maintain a stable pH level without controlling the ambient CO_2_ concentration. The number of cells was determined using a Neubauer counting chamber, and the final concentration of the cell suspension was adjusted to 10∙10^6^ cells ml^−1^. For microscopic examination, 500 cells mm^−2^ were seeded on the protein spotted glass slides and incubated for 15 min, 1 h or 24 h.

### SCORE

**Fig. 1:**
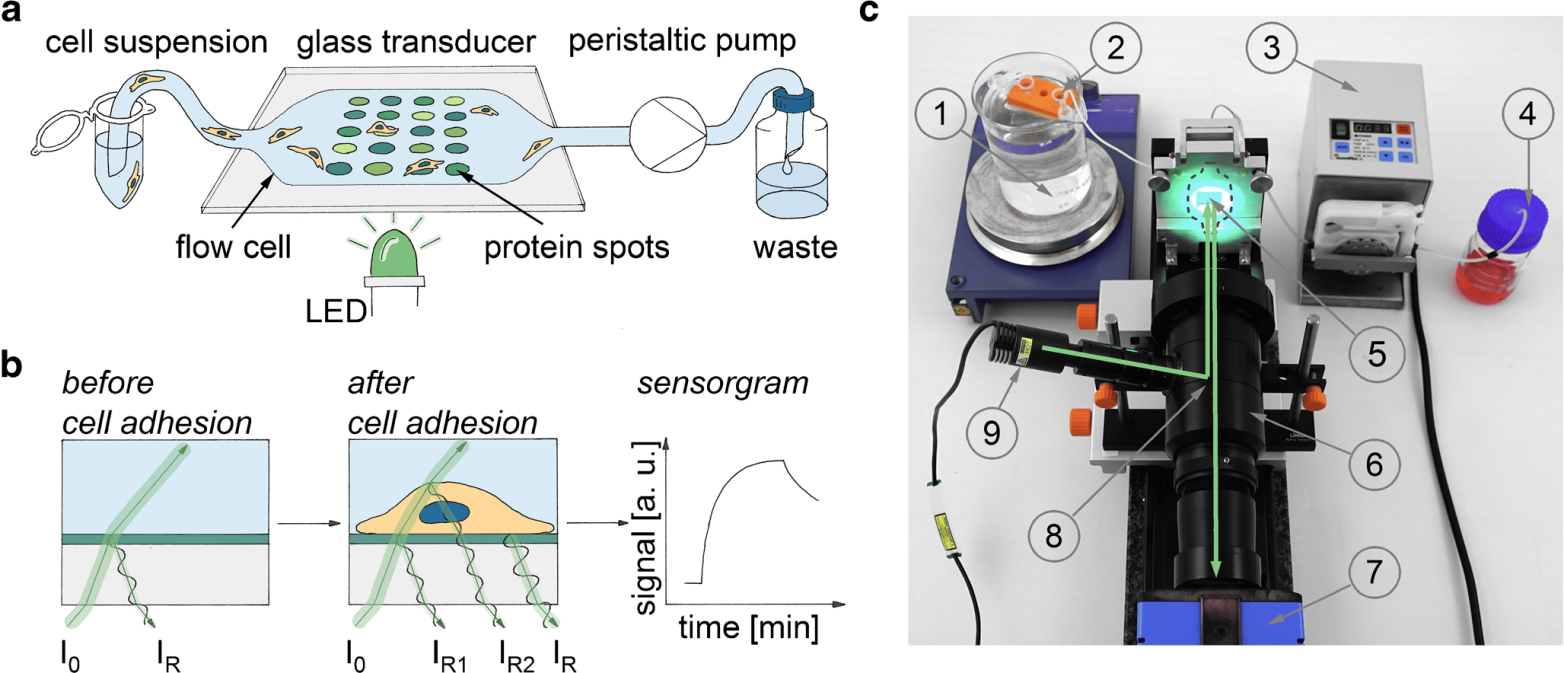
Schematic illustration of the SCORE measurement principle. **a** By use of a peristaltic pump, the transducer surface can be rinsed with different samples. The transducer with spatially distributed spots of different ECM proteins is illuminated by a green LED from the transducer bottom. **b** The green light (I_0_) is partially reflected (I_R_) at the interface between the glass transducer and the flow cell volume. The intensity of the reflected beam is collected via a CCD camera. Upon cell binding at the interface, a second partial beam is reflected at the interface between the cell and the surrounding liquid. The reflected beams (I_R1_, I_R2_) superimpose, resulting in an intensity change of the recorded reflected green light (I_R_). These changes in intensity are displayed over time in a sensorgram. **c** Real image of the SCORE setup. 1: water bath for temperature control, 2: sample, 3: peristaltic pump, 4: waste, 5: glass transducer with protein spots in the window area of the mount, 6: objective, 7: camera, 8: beam splitter, 9: LED. The green line illustrates the optical path of the setup. The dashed black line indicates the position of the flow cell

As described in [33], the transducer is illuminated perpendicularly using a green LED (530 nm) connected to a telecentric lens system, and the reflected light is detected via a CCD camera (2 mp) [34] at a rate of 1076 frame s^−1^ (Fig. 1a). A part of the green light is reflected at the glass/biolayer (protein coating) and a second partially reflected beam is reflected at the biolayer/liquid interface. Upon cell binding, the thickness of the biolayer is growing and the second partially reflected beam experiences a longer optical path. The partially reflected beams superimpose, modulating the captured light intensity (Fig. 1b). Images were recorded using the software CamWare (CamWareV3.00). ImageJ (ImageJ 1.48v) was used to edit and evaluate the captured images. The mean intensity of the reflected light was calculated for each protein spot at all timepoints. To compensate for illumination inhomogeneities, the signal change was determined by subtracting the intensity value right before cell injection and a normalization of the signal to the background signal at t = 15 min. The background signal was calculated using the mean signal intensity at t = 15 min of six circular areas symmetrically placed around the protein spot. The results for different protein spots are represented as sensorgrams. The intra-assay variation was calculated from the SCORE signal after 15-min cell adhesion time on four replicates on the transducer while the pooled data of three measurements with four replicates each was used for calculating the inter-assay variation.

The samples were pumped across the sensor surface with 0.37 ml∙min^−1^ using a peristaltic pump (Reglio Dig. MS/CA2-8C, tube diameter: 0.76 mm, Cole-Parmer GmbH, Wertheim, Germany) connected to the flow cell outlet. The cell suspensions and the cell culture medium were kept at a temperature of 37 °C throughout the entire measurement. First, a baseline using PBS was captured, followed by the injection of the cell culture medium to discriminate the later signal change caused by the adsorption of the cells from other components in the cell culture medium. Then, the cell suspension (10∙10^6^ cells ml^−1^ in Leibovitz’s L-15 Medium) was applied for 15 min. Adhesion and spreading of the cells were further evaluated injecting Leibovitz’s L-15 Medium for 45 min. Cells were detached using PBS for 15 min and trypsin/EDTA 5% for 10 min. To clean the setup, guanidinium hydrochloride (0.5%, pH 1.5) and PBS were used after each measurement, while ethanol (70%) and demineralized water were additionally used at the end of each measurement series.

### SCORE image processing

### Cell staining and microscopy

After incubation, cells were fixed for microscopic examinations in HISTOFIX (4%, P087.6, Carl Roth) for 15 min. Fixed cells were carefully washed with PBS and exposed to 1% Triton X (8059.0250, Th. Geyer, Renningen, Germany) for 15 min, followed by an incubation with AktinGreen (R37110, Thermo Fischer Scientific, Karlsruhe, Germany) in the dark for 30 min and by an incubation with NucBlue (R37605, Thermo Fischer Scientific, Karlsruhe, Germany) in the dark for 20 min. After washing with PBS, samples were covered with ProLong™ Gold Antifade Mountant (P10144, Thermo Fischer Scientific, Karlsruhe, Germany) and a cover glass and stored in the dark at 4 °C. Images were obtained by use of a Zeiss fluorescence microscope (Zeiss AXIO-OBSERVER, 1025388774, Zeiss).

### Microscope image processing

Counting the NucBlue-stained cell nuclei per area determined the number of adherent cells. The number of adherent MEF cells on the protein-coated spots was normalized to the number of adherent MEF cells on the bare glass substrate surrounding the protein spots. The size of the adherent cells was evaluated by measuring the area covered by the AktinGreen-stained F-actin.

### Statistical analysis

Results are presented as mean ± standard deviation. A one-way ANOVA (Levene’s test and Tukey test and with significance level of 0.05, 0.01, and 0.001) was performed using Origin 2021 (OriginLab, Northampton, MA, USA) for statistical analysis of the results.

## Results and discussion

### Cell adhesion measurements using SCORE

**Fig. 2:**
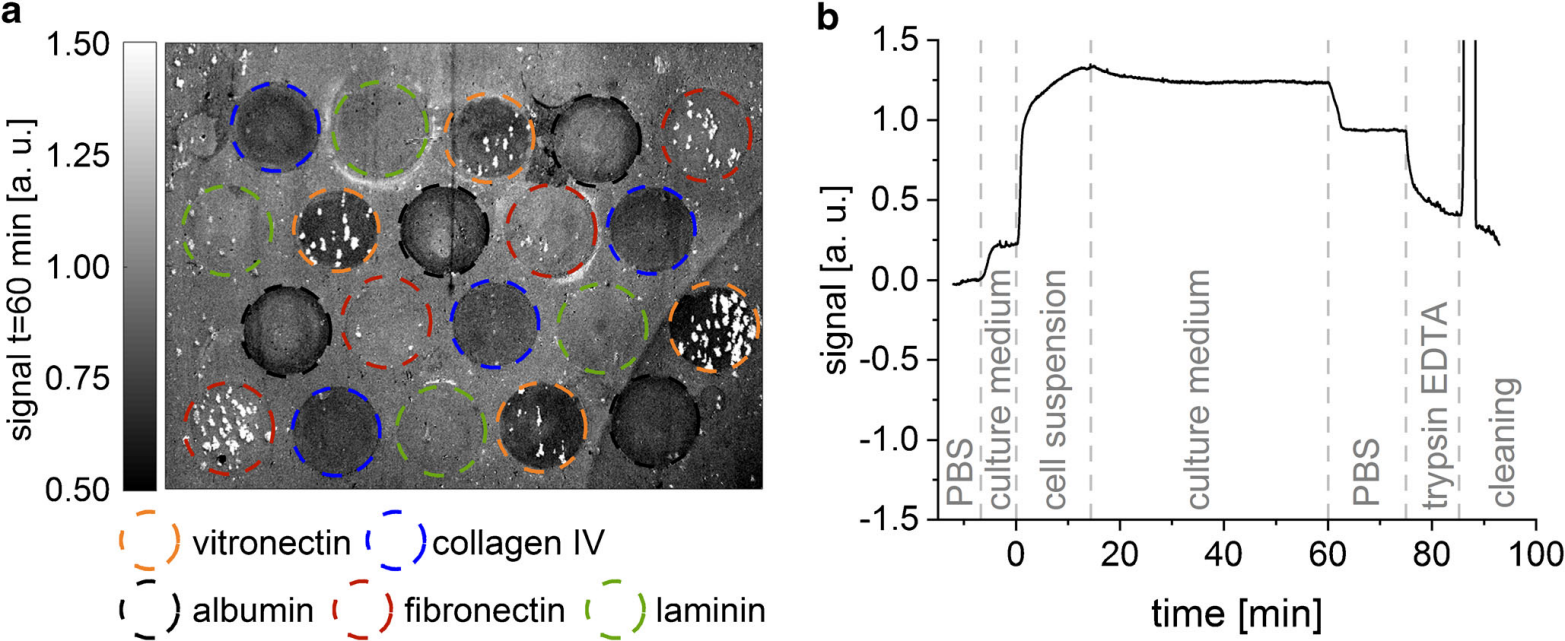
Typical results obtained by SCORE. **a** Image of the SCORE signal caused by the cell adhesion. The five different investigated proteins (four replicates each) were immobilized as indicated by the circles in the corresponding colour. **b** Binding curve of MEFs on a laminin-coated spot on the glass transducer. After 3-min baseline indicated by no signal change over time, the cell culture medium was pumped over the transducer surface for 8 min resulting in a small increase in signal indicating some non-specific adsorption of proteins in the culture medium. Since the signal reaches a plateau, it can be concluded the surface is completely blocked and further increase of the signal in the following steps is solely caused by adsorption of cells. Subsequently, the cell suspension was injected for 15 min leading to a major increase of the signal due to the adsorption of the cells to the immobilized proteins, followed by 45-min cell culture medium pumping over the transducer allowing the cells to spread or dissociate, the latter causing a small decay in signal. Cells were detached from the transducer surface by applying PBS for 15 min and trypsin/EDTA for 10 min resulting in a major decrease in signal indicating the detachment of cells and proteins. The cleaning of the setup was performed using guanidinium hydrochloride (0.5%, pH 1.5) and PBS

Slides with 20 protein spots with a diameter of 2 mm were prepared (Fig. 2a) and successfully measured in parallel. These initial SCORE measurements showed a sufficient signal-to-noise ratio and temporal resolution (Fig. 2b). Thus, SCORE fulfilled the above stated expectations and proved to be very suitable for monitoring the initial cell adhesion phase.

Before the injection of the cell suspension, the adhesion of other components of the culture medium reached a steady state. Thus, the following signal change during cell suspension injection is caused solely by the adhesion of the cells. Completing the measurement, the cells were detached from the surface applying PBS and trypsin/EDTA. The cell adhesion is strongly impaired by PBS and trypsin/EDTA, which is reflected in the SCORE measurements by an immediate signal decrease (Fig. 2b). Being able to change fluids during the measurement allows for the investigation of the effect of different reagents on the cell adhesion. This is an advantage of this setup compared to microtiter plate–based label-free systems like the EnSpire® Multimode Plate Reader with Corning® Epic® label-free technology.

**Fig. 3:**
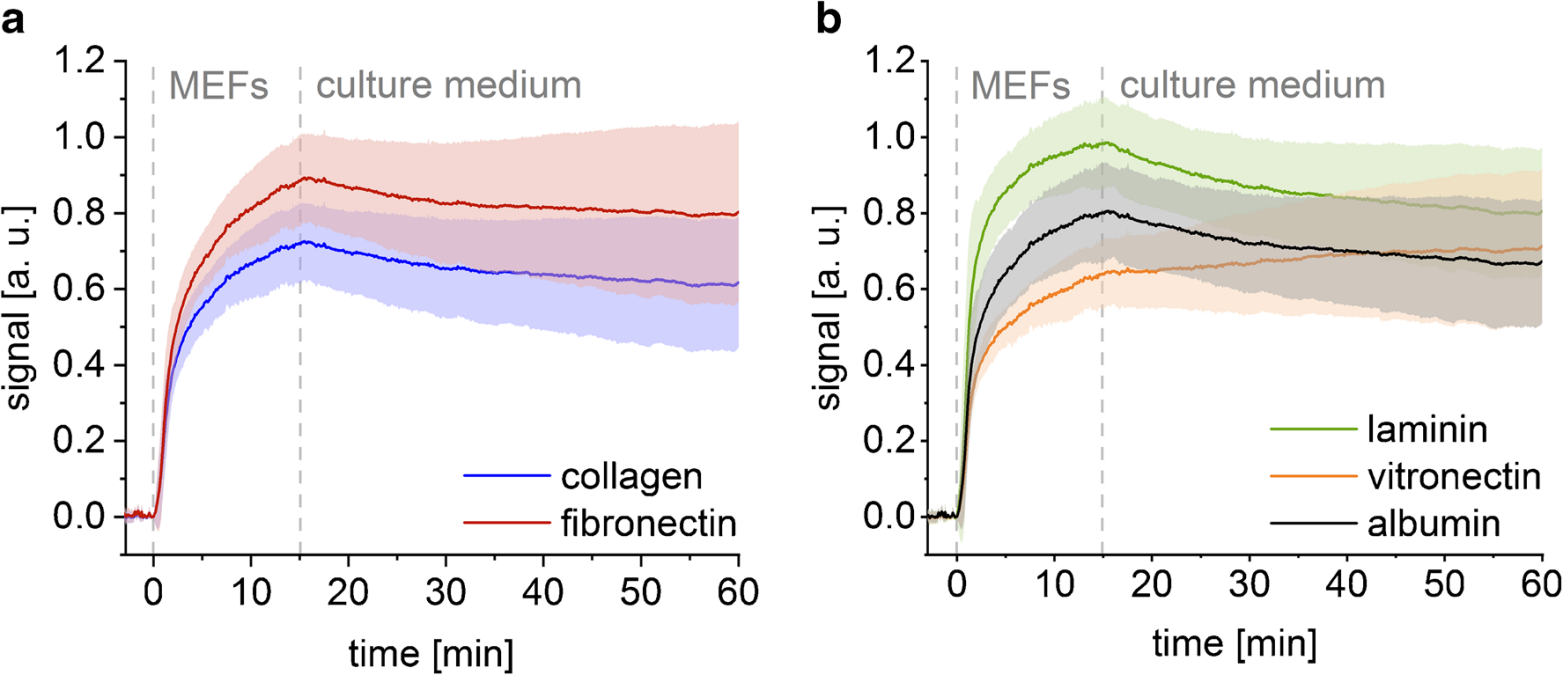
Binding curves of MEFs on glass spotted with collagen and fibronectin (**a**) and laminin, vitronectin, and albumin (**b**). The coloured areas illustrate the standard deviation at each timepoint of the mean (lines) of 12 replicates. The cell suspension was injected for 15 min, followed by the pumping of the cell culture medium across the transducer for 45 min

**Fig. 4:**
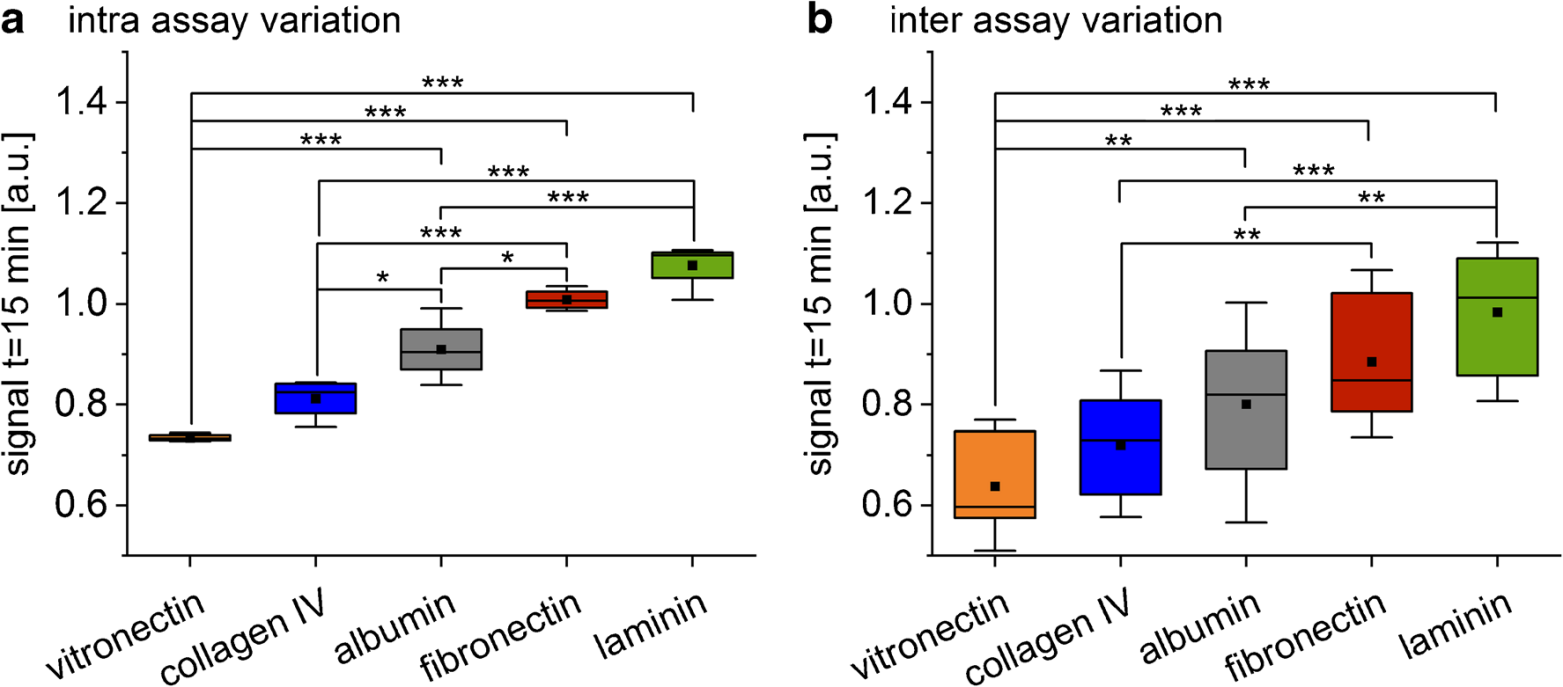
Comparison of **a** the intra-assay variation (n = 4) and **b** the inter-assay variation (n = 12). Box plot of the observed SCORE signal caused by MEF adhesion after 15 min on collagen-, fibronectin, laminin-, albumin-, and vitronectin-coated glass slides (One-way ANOVA, n = 12, *p < 0.05, **p < 0.01, ***p < 0.001). The means are illustrated by black dots and the medians by black bars. The coloured areas and the error bars represent the 25% and 75% quartiles and the 1.5 interquartile ranges, respectively

Comparing the SCORE signal after 15-min cell adhesion time, the largest signal change was found on laminin-coated glass. The other coatings showed decreasing signal intensities in the following order: fibronectin-, albumin-, collagen-, and vitronectin-coated glass (Figs. 3 and 4).

The time-resolved SCORE measurements also revealed differences in the adsorption kinetics of cell adhesion on different ECM proteins coated on glass. On laminin, albumin, and collagen, the signal change caused by the cell adhesion nearly reached an equilibrium surface loading, while on glass surfaces coated with vitronectin and fibronectin, the adhesion process was found to be slower, and the signal further increased at an adhesion time of 15 min (Fig. 3).

Desorption of cells during culture medium injection was found to be the most pronounced on glass surfaces coated with laminin, followed by glass surfaces coated with albumin and collagen. On fibronectin-coated glass, the cells were able to adhere slightly better; on vitronectin-coated glass, an increase in the signal was found throughout the entire desorption phase (Fig. 3). This phenomenon is later discussed in more detail under “Monitoring morphological changes of cells”.

To give a measure for the variation between the replica within one experiment and the reproducibility of the assay, the intra- and inter-assay variation was calculated, respectively. Variation of the cell adhesion within one experiment was considerably smaller compared to the differences between experiments (Fig. 4). This proves the great advantages of parallelized measurements in SCORE. When measuring cells, parallelization is particularly important as variations caused by using cells from different passages or phases in mitosis can be larger than the changes in cell behaviour caused by the experiment conditions. With this multiplexing approach, all investigated surfaces are measured at identical conditions at the same time and the variations caused by different states of the cells and sample preparation can be eliminated (Fig. 4a). Although, the intra-assay variations were smaller compared to the inter-assay variations, the presented assay showed a very good reproducibility and significant difference in the SCORE signal after 15-min cell adhesion time between many protein-coated glass surfaces (Fig. 4b).

A very simple and application-orientated coating procedure was applied. This causes inhomogeneities in the protein-coated areas (Fig. 2a). Furthermore, adhesion of the cells was expected on all surfaces. Despite these difficult experimental conditions, significant differences in the cell adhesion on the surfaces were found using SCORE. The good reproducibility and the possibility to discriminate even minor differences in the adhesion behaviour of cells is ensured by the multiplexing and the relatively large spot area (diameter of 2 mm) averaging the adhesion behaviour of many cells.

### Dynamics of the initial cell adhesion

**Fig. 5:**
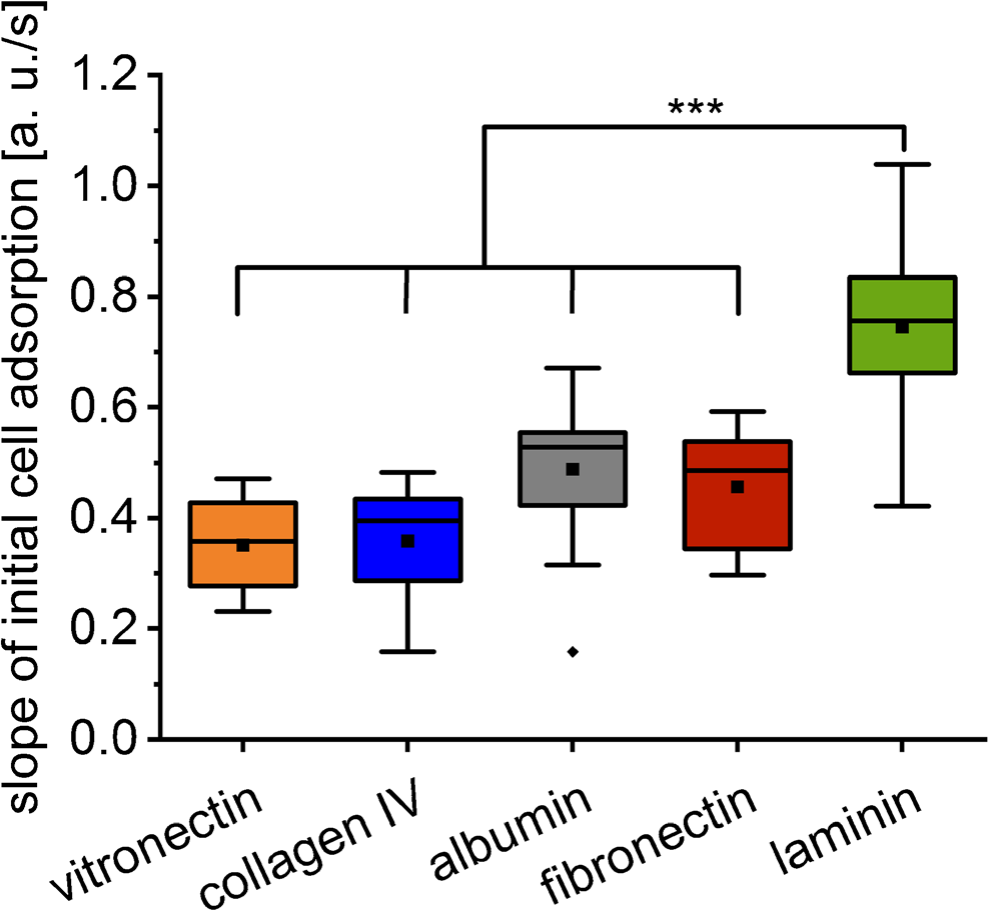
Box plot of the slope of the adhesion curve measured by SCORE caused by the initial cell adhesion on collagen-, fibronectin-, laminin-, albumin-, and vitronectin-coated glass slides (one-way ANOVA, n = 12, ***p < 0.001). The means are illustrated by black dots and the medians by black bars. The coloured areas and the error bars represent the 25% and 75% quartiles and the 1.5 interquartile ranges, respectively

As mentioned, a crucial parameter when investigating cell adhesion processes is the initial speed which partially reflects the kinetics of the cell adhesion. Therefore, this initial binding was evaluated in detail. In the present study, the slope during the initial cell adhesion—within the linear range of the adsorption curve—is significantly higher on laminin-coated glass surfaces compared to the other surfaces (Fig. 5). On this surface also, the SCORE signal change after 15 min was found to be the highest (Fig. 4). The adhesion of cells on fibronectin-coated glass surfaces on the other hand caused a large SCORE signal change after 15 min while showing a rather slow initial adhesion speed (Figs. 4 and 5).

These observations proved our expectations that the adsorption kinetics of cells differ on different ECM proteins. Combining the SCORE signal after 15 min, the monitored dissociation and the initial adsorption speed, a comprehensive description of the cell adhesion process is achieved. These differences in the adsorption behaviour of the cells on the different surfaces are summarized in Table 1. When using other—non-time-resolved—methods, this valuable information would have been lost.

**Table 1:** Summary of the adhesion behaviour of MEF cells on different ECM protein–coated glass surfaces

	Initial binding [a. u./s]	Signal after 15 min [a. u.]	Equilibrium reached	Dissociation
Vitronectin	0.35 ± 0.08	0.64 ± 0.09	No	Signal increase
Collagen IV	0.36 ± 0.10	0.72 ± 0.11	Nearly	Medium
Albumin	0.49 ± 0.14	0.80 ± 0.13	Yes	Large
Fibronectin	0.46 ± 0.11	0.89 ± 0.12	No	Small
Laminin	0.75 ± 0.15	0.98 ± 0.12	Yes	Very large

### Fluorescent imaging of adherent cells

To verify the initial promising SCORE results, fluorescence microscopy was performed to correlate the SCORE signal after a cell adhesion time for 15 min with the behaviour on a cellular level.

**Fig. 6:**
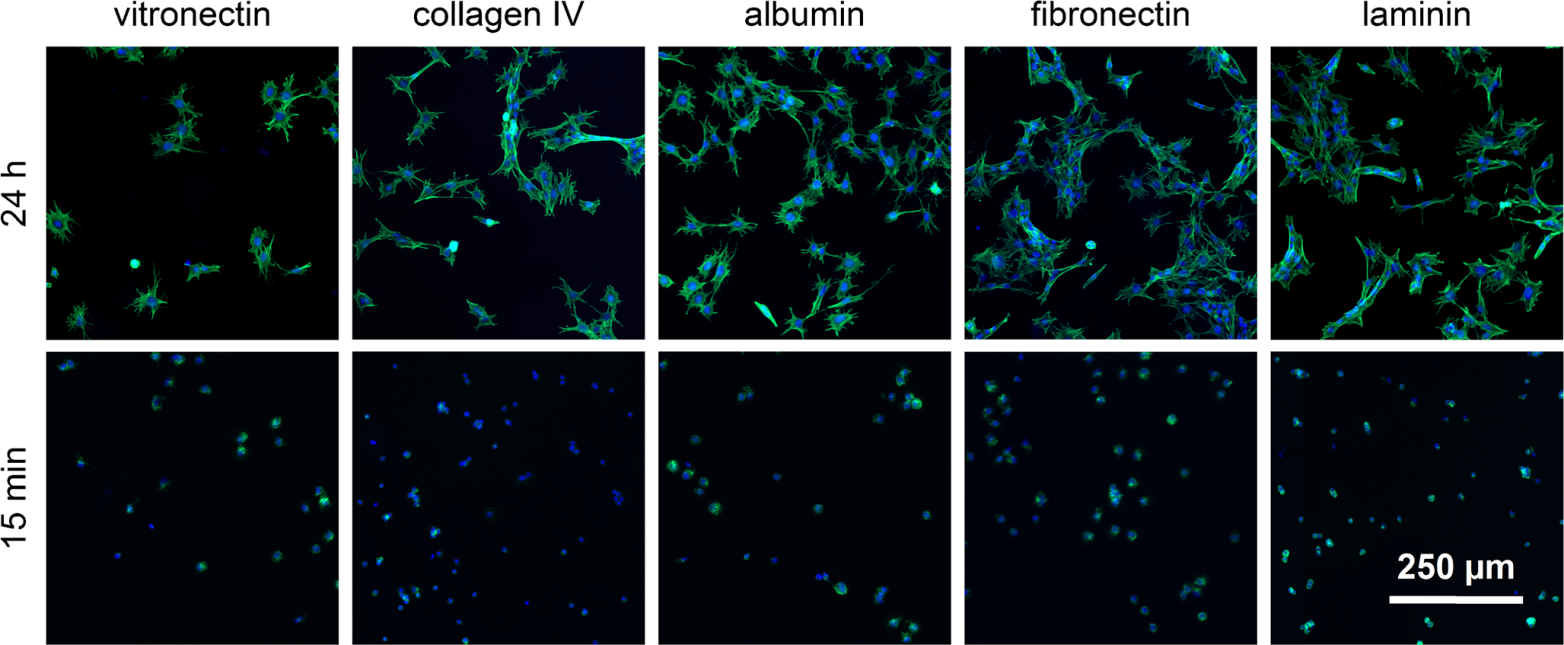
Morphological examination of adherent MEF cells on protein-coated glass slides. The green-stained F-actin displays the cytoskeleton organization at 15-min and 24-h adhesion time. The number of blue-stained nuclei reflects the number of adherent cells

The different surfaces did not appear to influence the morphology of the MEF cells after 15-min adhesion time (Fig. 6). After incubation for 24 h, cells adhered and spread on all protein-coated glass slides (Fig. 6).

**Fig. 7:**
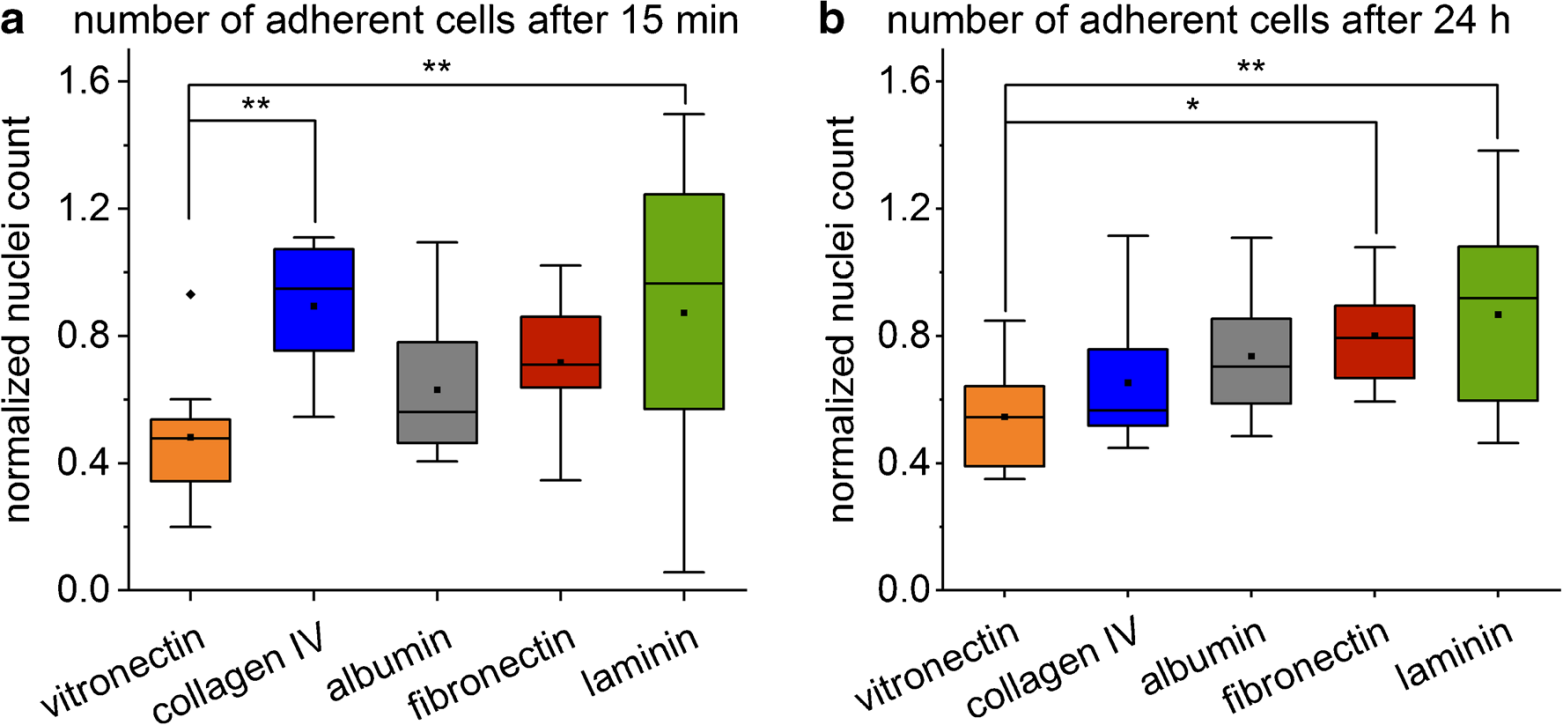
Box plot of the normalized nuclei count of adherent MEFs after 15 min (**a**) and 24 h (**b**) on collagen-, fibronectin-, laminin-, albumin-, and vitronectin-coated glass slides (one-way ANOVA, n = 12, *p < 0.05, **p < 0.01). The means are illustrated by black dots and the medians by black bars. The coloured areas and the error bars represent the 25% and 75% quartiles and the 1.5 interquartile ranges, respectively

The number of adherent cells showed minor differences between the surfaces (Fig. 7). A slight increase in the number of adherent cells from vitronectin- to collagen IV-, albumin-, fibronectin- to laminin-coated glass slides occurred after an incubation of 24 h. A significant difference can be observed between vitronectin and fibronectin as well as between vitronectin and laminin (Fig. 7b). After incubation for 15 min, the number of adherent cells on all surfaces was similar to the number of adherent cells after incubation for 24 h, except on collagen IV–coated glass slides. The uncertainties of fixing and staining cells after a short adhesion time of 15 min is reflected in the larger variance in the normalized cell count (Fig. 7a). Additionally, the process of fixing and staining the cells requires several exchanges of liquids which might wash off weak bound cells and thus increase the deviation even further.

### Correlation between the SCORE signal and the number of adherent cells

To compare the SCORE signal after 15 min, when the cell already adhered to the surface but no change in the cell morphology occurred [35], to the number of adherent cells, fluorescent microscopic images of adherent cells after 24 h were used. The later timepoint was chosen as the nuclei counting was more robust while the possible influence of proliferation was still minor.

**Fig. 8:**
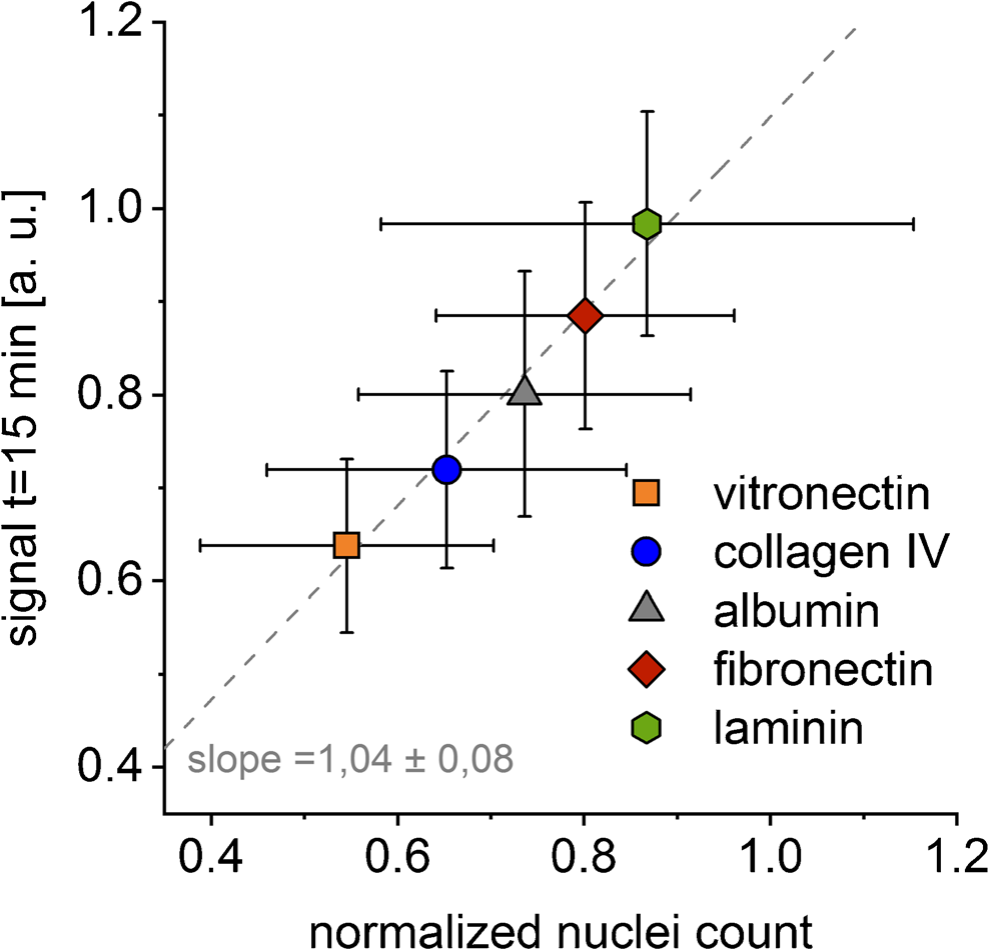
Linear correlation between the SCORE signal after 15-min adhesion time and the number of fixed and stained adherent cells after 24 h on protein-coated glass slides

The excellent correlation between the SCORE signal and the number of adherent cells after 24 h (Fig. 8) proves that these results obtained by using this direct optical method reflect the cell binding processes at the transducer surface. Choosing the SCORE settings and experiment setup presented here, the intensity change upon cell binding events at the transducer surface is within the linear working range of SCORE.

Interestingly, SCORE measurements deliver the results after a cell adhesion of 15 min under flow-through conditions, while cells for microscopic examination need to be cultured for 24 h, followed by image taking and an elaborate image processing.

The variances in SCORE measurements were smaller compared to microscopic examination of fixed and stained cells, showing that cell adhesion measurements using SCORE are very robust. Error-prone pipetting, fixation, and staining steps are not required. Also, using sophisticated fluidics ensures that cells are homogeneously distributed over the entire investigated area.

### Monitoring morphological changes of cells

After the cells initially adsorb to the surface, internal signal cascades initiate the attachment of the cells to their environment, followed by the cytoskeleton remodelling leading to the spreading of the cells at the surface. These morphological differences cause changes in the surface area covered by the cell, and in the refractive index of the cell cytoplasm [36, 37]. This, in turn, influences the optical path of the captured reflected light in SCORE. Thus, the cell spreading phase in cell adhesion can also be monitored by SCORE. Contrary to evanescent field techniques as, e.g. SPR or grating couplers, in which the signal change is caused by refractive index changes in close vicinity to the transducer surface, the signal change in SCORE is influenced by the size of the cell and the cytoskeleton reconstruction inside the entire cell.

**Fig. 9:**
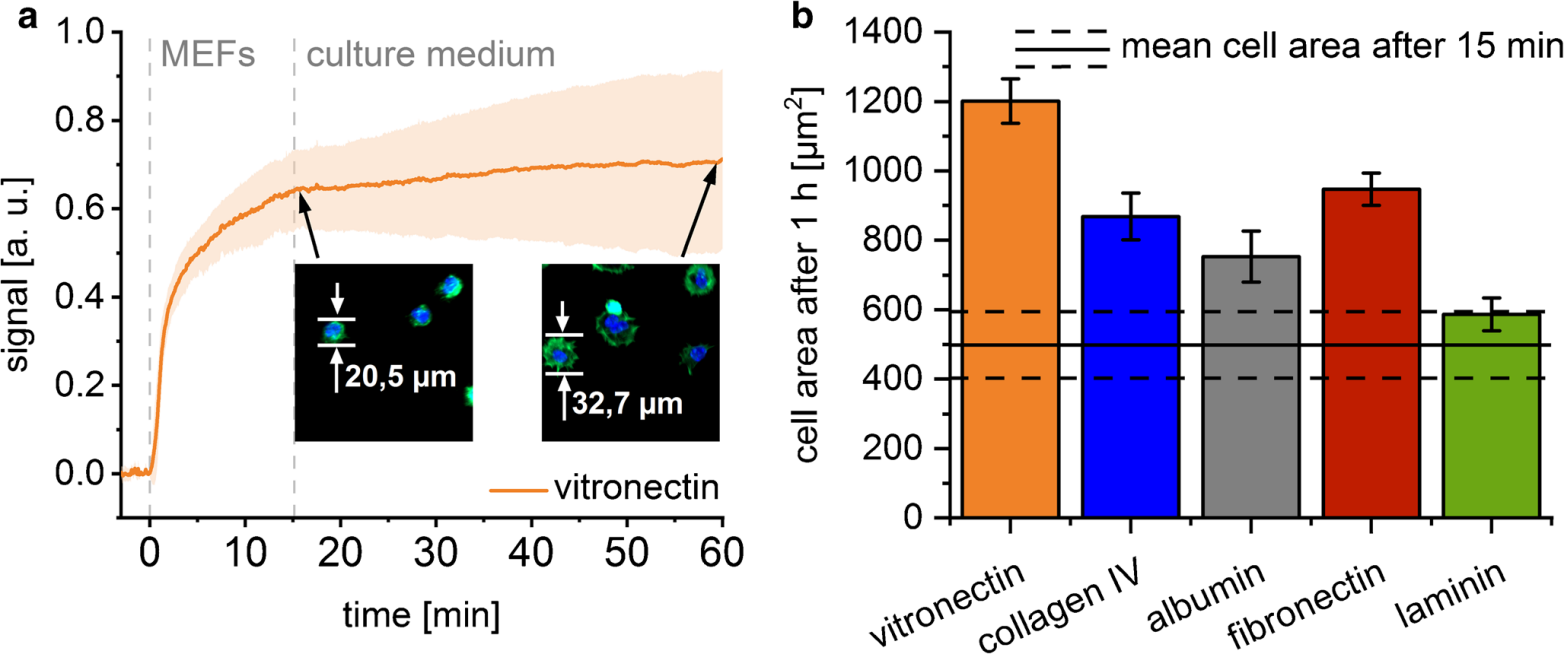
Influence of the cell spreading on the signal change measured with SCORE. **a** Sensorgram of MEF cell adhesion on vitronectin-coated glass (orange line). The morphology of the cells is illustrated by typical fluorescent microscopy images. **b** The size of the adherent MEF cells after an adhesion time of 1 h (n = 4) on glass coated with different proteins (bars) in comparison to the cell size after 15 min (black line)

Within the first 15 min, the morphology of the adherent cells is not influenced by the different substrates. This results in the excellent correlation between the SCORE signal and the number of adherent cells. However, deviations from this correlation are expected when morphological changes of the cell occur over time. On glass coated with vitronectin, the SCORE signal of the adherent cells shows a steady increase during rinsing with cell culture medium. Since the number of cells on the surface cannot increase during this time, the signal increase must be caused by the spreading of the cell on the transducer surface. This is verified by the microscopic images taken after 15-min and 1-h adhesion time (Fig. 9a).

The investigated cells showed different morphologies on different surfaces. This was measured by calculating the size of the adherent cells, reflecting the degree of spreading (Fig. 9b). On vitronectin-coated surfaces, the cells showed the biggest size followed by the surfaces coated with fibronectin. The smallest increase in cell size after an adhesion time of 1 h compared to an adhesion time of 15 min was found on laminin-coated surfaces (Fig. 9b). However, a decrease in the SCORE signal is recorded during cell culture medium injection on all surfaces with the exception of vitronectin (Fig. 3). This is either due to the concurrent desorption of weakly bound cells leading to an overall signal decrease, or the delayed spreading of the cells on collagen-IV-, albumin-, fibronectin-, or laminin-coated glass substrates outside the investigated time period.

Therefore, SCORE proved to be an excellent tool to study the entire cell adsorption process including morphological changes on different substrates in real time.

## Conclusion and outlook

The results demonstrate that the entire adhesion process of cells to different ECM protein–coated surfaces could be successfully monitored using SCORE in a parallelized, label-free, and time-resolved manner. Remarkably, results obtained by SCORE measurements were more robust compared to the results obtained from microscopic fluorescent imaging of fixed and stained adherent cells. Due to the multiplexing possibility in SCORE, the adhesion of cells to 5 different surfaces—with four replicates each—was measured in real time in parallel with improved comparability between the surfaces and good reproducibility. In SCORE, the multiplexing scale up is just limited by the utilized optics determining the observable area. Thus, the number of different spots on the transducer can easily be increased further and many more than the here presented 20 spots can be measured simultaneously at defined conditions instead of sequential measurements. Further enlargement of the size of the spots or the number of replicates would enable averaging the behaviour of more cells giving even more robust results. Also, the measurement of single cell adhesion seems feasible when incorporating optics with a larger magnification. The high time resolution of SCORE allowed for the discrimination between different adhesion phases of the cells and revealed details about the adhesion speed and the spreading characteristics of the cells on different surfaces. As a result, a comprehensive picture of the cell adhesion is gained. Thus, applicability for the investigation of competitive binding behaviour to a large number of possible ligands as well as to their mixture is possible. Using sophisticated fluidics in the SCORE setup, it was possible to measure the cell adhesion under precise flow conditions, and an easy exchange of sample fluids during the measurements is possible which is required for many cell-based sensing applications. Especially, the investigation of distinct signalling cascades during the foreign-body reaction on an implant surface (e.g. immune responses and thrombogenesis) by selective receptor knock-out or screening tasks in effect-based analytics are of high interest.

Considering the embedded microfluidics, the label-free optical principle, the high time resolution, the robustness against temperature fluctuations, the large penetration depth, and the multiplexing possibility, SCORE proved to be an excellent method for studying the cell adhesion to biomaterials and provides additional and complementary levels of information compared with other state-of-the-art methods.
